# Identification of core objectives for teaching sustainable healthcare education

**DOI:** 10.1080/10872981.2017.1386042

**Published:** 2017-10-12

**Authors:** Arianne Teherani, Holly Nishimura, Latifat Apatira, Thomas Newman, Susan Ryan

**Affiliations:** ^a^ Department of Medicine and Center for Faculty Educators, School of Medicine, University of California, San Francisco, CA, USA; ^b^ Center for Faculty Educators, School of Medicine, University of California, San Francisco, CA, USA; ^c^ Occupational Health Services, The Permanente Medical Group, South San Francisco, CA, USA; ^d^ Departments of Epidemiology and Biostatistics and Pediatrics, School of Medicine, University of California, San Francisco, CA, USA; ^e^ Department of Anesthesia, School of Medicine, University of California, San Francisco, CA, USA

**Keywords:** Medical education, climate change, objectives, Delphi, sustainable healthcare education

## Abstract

**Background**: Physicians will be called upon to care for patients who bear the burden of disease from the impact of climate change and ecologically irresponsible practices which harm ecosystems and contribute to climate change. However, physicians must recognize the connection between the climate, ecosystems, sustainability, and health and their responsibility and capacity in changing the status quo. Sustainable healthcare education (SHE), defined as education about the impact of climate change and ecosystem alterations on health and the impact of the healthcare industry on the aforementioned, is vital to prevention of adverse health outcomes due to the changing climate and environment.

**Objective**: To systematically determine which and when a set of SHE objectives should be included in the medical education continuum.

**Design**: Fifty-two SHE experts participated in a two-part modified-Delphi study. A survey was developed based on 21 SHE objectives. Respondents rated the importance of each objective and when each objective should be taught. Descriptive statistics and an item-level content validity index (CVI) were used to analyze data.

**Results**: Fifteen of the objectives achieved a content validity index of 78% or greater. The remaining objectives had content validity indices between 58% and 77%. The preclinical years of medical school were rated as the optimal time for introducing 13 and the clinical years for introducing six of the objectives. Respondents noted the definition of environmental sustainability should be learned prior to medical school and identifying ways to improve the environmental sustainability of health systems in post-graduate training.

**Conclusions**: This study proposes SHE objectives for the continuum of medical education. These objectives ensure the identity of the physician includes the requisite awareness and competence to care for patients who experience the impact of climate and environment on health and advocate for sustainability of the health systems in which they work.

**Abbreviations**: CVI: Content validity index; SHE: Sustainable healthcare education

## Introduction

Climate change and ecosystems degradation present the ‘greatest threat’ to public health in this century [1–3]. Physicians will be called upon to care for patients who bear the burden of disease from the impact of climate change and ecologically irresponsible practices which harm ecosystems and contribute to climate change. Many diseases and health burdens are linked to climate fluctuations including respiratory illness, infectious diseases, and malnutrition [4]. Furthermore, physicians work within the wasteful, high eco-footprint healthcare system, which has barely begun to embrace a culture of sustainability [5]. Physicians are in a position to view sustainability from multiple angles, to move the health and healthcare culture toward greater ecological responsibility and, as a consequence, improve patient and public health. The latter position reflects the physician’s identity as one of an advocate to ‘promote those social, economic, educational, and political changes that ameliorate the suffering and threats to human health’ [6,7]. However, physicians must first recognize the connection between the climate, ecosystems, sustainability, and health and their responsibility and capacity as health professionals in changing the status quo [8].

Described by the Sustainable Healthcare Education Network, ‘sustainable healthcare education’ (SHE) is education about the impact of climate change, ecosystem alteration, and biodiversity loss on health as well as the impact of the healthcare industry on the aforementioned [9,10]. Nomenclature related to SHE has included ‘environmental sustainability’ [11], ‘ecosystems and health’ [12], ‘ecosystem health’ [13], ‘climate change environment degradation, biodiversity and health’ [14], and ‘environmental accountability’ [15]. There is currently little SHE in medical education curricula [12,14]. The health impacts of environmental change will be experienced by all of society albeit unequally, with those least responsible for the change (e.g., children, the world’s poor) affected the most [10]. Socially accountable education emphasizes the use of education, research, and service to address health concerns through approaches that engage interdisciplinary professionals, organizations, and the public [16,17]. A SHE curriculum developed from within the framework of social accountability provides a critical scaffold for students and teachers to understand the importance of what is learned to the healthcare needs of the patients they serve. Moreover, a SHE curriculum resonates with aims [18] for bettering the healthcare system by (1) improving population health through proactive anticipation of society’s healthcare needs and attention to prevention, and (2) reducing healthcare costs by focusing on the sustainability and resource efficiency (including containment of waste and cost) in the healthcare system [5].

Currently few medical schools offer electives, some student-run, that focus on the impact of climate change on health and/or creating sustainable healthcare practices [5]. A recent review found that medical students and physicians know about ecosystems but need more education on causes and consequences of environmental change [12]. The Sustainable Healthcare Education Network developed a representative set of learning objectives [10] to guide both undergraduate and graduate medical education in SHE, grouped into three priority learning areas:

1. Describe how the environment and human health interact at different levels.
2. Demonstrate the knowledge and skills needed to improve the environmental sustainability of health systems.
3. Discuss how the duty of a doctor to protect and promote health is shaped by the dependence of human health on the local and global environment.

Little is known about which SHE objectives are core and when in the continuum of medical education core objectives should be introduced. Moreover, as knowledge proliferates, the demands increase for what learners should know to become physicians [19]. Today the undergraduate and graduate medical education curriculum is crowded with content learners must know. Systematically developed SHE objectives are needed to guide medical educators to prioritize what they teach across the continuum of the crowded medical education curriculum. Ultimately, a SHE curriculum will provide physicians with the necessary awareness, knowledge, and skills to care for patients who experience the impact of climate and environment on health and advocate for the sustainability of the health systems in which they work. The aim of our study was to provide guidance on which and when a set of SHE objectives should be included in the continuum of medical education.

## Methods

### Design and setting

We used a modified Delphi approach to conduct a two-step survey of SHE experts between June and October 2015. The University of California, San Francisco (UCSF) institutional review board approved the study as exempt.

### Participants

We surveyed physicians and academics who had expertise or engaged in one or more of the following activities around the topics of climate change, environmental literacy, environmental and/or ecosystem health, or healthcare sustainability [20]: (1) research, (2) writing/publishing, (3) teaching, (4) activism, or (5) administration.

Respondents consisted of experts identified through (1) a literature search, (2) web search for individuals working in relevant organizations and the community such as the state departments for public health, Physicians for Social Responsibility, Health Care without Harm (to ensure we held the principles of social accountability described earlier), and (3) snowball sampling. Snowball sampling techniques, in which respondents are asked to solicit other experts, ensured that as many expert perspectives as possible were represented [21].

### Objectives and survey

Our survey consisted of a set of SHE objectives created in two distinct phases. During the first phase (2009), an education sub-committee, representing the professions at UCSF (Medicine, Dentistry, Nursing, and Pharmacy) and interest in SHE was charged by the UCSF Academic Senate Sustainability Committee. Based on a literature and web search, the sub-committee created a comprehensive set of SHE learning objectives for UCSF health professions learners. One committee member (AT) extracted articles with no start date through 2009 via PubMed and CINAHL in English using the search terms ‘ecosystems’, ‘climate change’, ‘environment’, ‘sustainability’, ‘environmental sustainability’, ‘health’, and ‘education’ with Boolean operators. Reference lists of identified manuscripts located additional articles. Articles were included regardless of type. The committee member used the same terms to search for institutions whose focus was on SHE education. Another committee member (TN) drafted objectives. All committee members reviewed and revised the objectives. This process resulted in 30 SHE objectives.

In the second phase (2015), the investigators consulted the priority learning outcomes developed by the Sustainable Healthcare Education Network [10,22], a collaboration of academics, physicians, and healthcare students. The Network created objectives through a structured feedback process from all medical schools, Royal colleges, post-graduate deaneries, and major medical organizations in the United Kingdom [10]. These learning objectives were a representative set meant to guide undergraduate and graduate medical education. Our aim was to be comprehensive. Hence, where Sustainable Healthcare Education network and UCSF objectives aligned, we used the former’s 13 objectives. We then augmented the list by including additional eight UCSF objectives. The initial mapping of the objectives was completed by one author (AT). Subsequently three investigators (LTA, TN, SR) reviewed mapping of the two sets of objectives and organization by the Sustainable Healthcare Education Network’s priority areas (see Introduction). Discrepancies were discussed and consensus reached on the final objectives and their placement. The resulting 21 objectives were used in the survey.

The survey asked respondents to provide demographic information and description of their SHE expertise. Respondents independently rated the importance of each objective using a 1 to 4 scale (1 = not very important, do not include; 2 = moderately important; 3 = important; 4 = very important); and when in training or the continuum of medical education (1 = premedical school; 2 = preclinical years of medical school; 3 = clinical years of medical school; and 4 = postgraduate years (e.g., residency, fellowship)) should a learner be taught this objective. To provide context (i.e., experiences with and perception of the objectives) to ratings, at the end of the survey we asked respondents to describe via open-ended questions if and how their institution addressed the objectives. We distributed the surveys via Qualtrics^TM^ accompanied by an information sheet describing the modified Delphi procedure.

### Modified Delphi procedure

One of the steps in curriculum development involves establishing content validity of content [23–25]. This step determines whether the content measures the construct (i.e., core SHE knowledge) for the intended population (i.e., learners in the medical education continuum). To conduct a content validation of the objectives we conducted a modified-Delphi procedure. The Delphi technique is typically used to gather a reliable opinion from a group of experts via sequential surveys, including quantitative feedback on prior responses [26]. In a typical modified-Delphi study experts complete a first round of ratings and are asked to complete a second round in which they are given the round 1 ratings of all the experts to allow them to reconsider their responses informed by information received from other experts [26,27]. Respondents rated the importance and level of education for the objectives in round 1. In round 2, all original respondents including those identified during the snowball sample were re-surveyed. In this re-survey respondents were provided with their own individual round 1 ratings and all respondents’ distribution of ratings to inform their round 2 responses. For the snowball sample, 19 (47.5%) of the 40 experts (see Results section for response rate details) who responded to the first round recommended additional experts. Thirteen (68.5%) of the 19 respondents recommended between one and five experts and six (31.5%) recommended between six and 13 experts. The list of recommended experts was subsequently examined to determine which ones were not already surveyed or recommended by multiple experts.

### Analysis

Respondents’ demographics were displayed using descriptive statistics. For each objective, we calculated an item-level content validity index (CVI) from the second round of ratings [28]. A content validity index is used to quantify the relevancy of objectives and provides information about the proportion of respondents in agreement with relevance of each objective [29]. For adequate content validity, a CVI of .78 or greater has been recommended in the literature [29]. We considered objectives as having sufficient content validity if 78% or more (CVI = .78 or greater) of the respondents rated them as a 3 (important) or 4 (very important) [27]. To determine whether the Delphi method impacted the ratings between rounds, we examined whether the mean variance changed between the two rounds. Level of education was analyzed using descriptive statistics. Open-ended questions were analyzed by one investigator (AT) using the qualitative content analysis [30]. The investigator first read through all responses to the open-ended questions and through an open coding process [31] generated an initial list of codes. The investigator then applied the list of codes to all the responses. The investigator discussed coding uncertainties with an additional investigator (SR) and finalized coding through discussion.

## Results

We sent the survey to 50 experts of whom 40 (80%) responded, and subsequently to 32 experts identified through the snowball sample of whom 12 (37.5%) responded. In total, 52 of 82 (63.4%) experts completed the surveys in both rounds. Table 1 displays participants’ demographic data. Most respondents were from the United States, physicians, and affiliated with a public university. Respondents ascribed their expertise to multiple areas of which research was most prominent.

**Table 1.** Demographic characteristics of 52 experts participating in a modified Delphi survey of sustainable healthcare education (SHE) objectives for inclusion in medical education.

Characteristic	Category	Mean (%)
Country	United States	40 (76.9)
	United Kingdom	8 (15.4)
	Australia	2 (3.8)
	Canada	1 (1.9)
	India	1 (1.9)
Profession	Physicians	29 (55.8)
	Physicians with additional degree (e.g., MPH)	10 (19.2)
	PhD	11 (21.2)
	Other (e.g., MPH, MSW)	2 (3.8)
Affiliation	Public university	26 (50)
	Private university	15 (28.8)
	Health system	4 (7.7)
	Non-profit organization	4 (7.7)
	Government organization	3 (5.8)
SHE expertise^a^	Research	26 (50)
	Activism	21 (40.4)
	Teaching	14 (26.9)
	Administration	10 (19.2)
	Writing/publishing	6 (11.5)

^a^Respondents were allowed to select multiple categories

Table 2 shows the mean ratings, CVI, and modes for training time period in training for the proposed objectives. Fifteen of the objectives achieved a CVI of 78% or greater. Of these fifteen, three objectives received CVIs of 90% or greater. The objectives with CVI of 78% or greater were part of all priority areas and included every objective in area 1 (how the environment and human health interact at different levels). Six objectives had CVIs between 58% and 77%. Of these six objectives, 3 received CVIs less than 70%. The average variance for round 1 ratings were .67 which remained stable through the second round at .68, indicating that participants didn’t change their ratings much between rounds. The preclinical years of medical school were rated as the appropriate time for introducing 13 of the objectives, and the clinical years were rated as the optimal time for introducing six of the objectives. A majority of the respondents felt that learners should learn the definition of environmental sustainability prior to medical school and identify ways to improve the environmental sustainability of health systems in post graduate training.

**Table 2.** Mean rating, content validity index (CVI), and mode of time period in training for proposed sustainable healthcare education (SHE) objectives as rated by 52 experts during a modified Delphi procedure.

Objective	Mean rating^a^ (SD)	CVI for ratings of 3 or 4 %^b^	Mode for training time period^c^
**Describe how the environment and human health interact at different levels** (*Doctor as scholar and scientist)*			
Outline the dependence of human health on global and local ecological systems, which supply essentials such as air, water, and a stable climate	3.75 (.52)	96	2
Describe the mechanisms by which human health is affected by environmental change, for example through changes in disease vectors, exposure to extreme weather, migration, and reduced food security.	3.73 (.53)	96	2
Describe features of a health-promoting local environment, in community and healthcare settings, to include access to green spaces, clean air, and an active travel infrastructure	3.46 (.67)	90	2
Discuss the contribution of human activity and population size to global environmental changes such as climate change, biodiversity loss, and resource depletion	3.42 (.79)	87	2
Explain the concept of environmental justice and the core principles for addressing it	3.25 (.97)	81	2
Discuss medical, ethical, legal, and economic factors in caring for patients with environmental disease	3.25 (.86)	81	3
**Demonstrate the knowledge and skills needed to improve the environmental sustainability of health systems (***Doctor as practitioner)*			
Identify ways to improve the environmental sustainability of health systems – in individual practice, in health service management, and in the design of care systems	3.38 (.80)	88	4
Identify potential synergies between policies and practices that promote environmental sustainability and those that promote health	3.34 (.90)	87	2
Define environmental sustainability	3.33 (.79)	85	1
Take a focused occupational and environmental history	3.33 (.73)	85	3
Explain how trends in demographics, technology, climate, and resource availability may affect our ability to provide healthcare into the future	3.13 (.90)	79	2
Describe, with examples, the different types of environmental impact resulting from healthcare provision, and how these may be measured	3.17 (.81)	79	3
Diagnose, treat, and/or prevent adverse health effects to attributable to global climate change or environmental causes (e.g., illness from extreme weather conditions, disease vectors, inspired air pollutants - in particular ozone, particulate matter, and/or ingested pollutants from food or water)	3.17 (.90)	75	3
Explain bioaccumulation and biomagnification of pollutants	2.92 (.93)	69	2
Identify patients most vulnerable to climate change and advise them accordingly	2.88 (1.00)	63	3
**Discuss how the duty of a doctor to protect and promote health is shaped by the dependence of human health on the local and global environment**. (*Doctor as professional)*			
Discuss ethical tensions between allocating resources to individual patients and protecting the environment upon which the health of the wider community depends	3.25 (.72)	83	2
Evaluate their work environment for level of sustainability	3.06 (.78)	81	3
Explain how the health impacts of environmental change are distributed unequally within and between populations and the disparity between those most responsible and those most affected by change	3.21 (.82)	79	2
Discuss competing interests within healthcare (cost, infection control, safety) contributing to environmental inefficiency	3.23 (.85)	77	2
Recognize and articulate personal values concerning environmental sustainability, given the relationship between the environment and the health of current and future generations	3.00 (.79)	73	2
Demonstrate awareness of organizational sustainability policies and the legal frameworks for reducing carbon emissions	2.79 (.87)	58	2

^a^Scale: 1 = not important; do not include, 2 = moderately important, 3 = important, 4 = very important.

^b^The CVI represents respondents who rated this objective as 3 (important) or 4 (very important).

**^c^**Scale: 1 = prior to medical school, 2 = preclinical years of medical school, 3 = clinical years of medical school, 4 = postgraduate years

On the open-ended questions, nineteen respondents stated that their institution or workplace explicitly addressed some SHE objectives. These objectives were addressed primarily in the preclinical medical school curriculum with some institutions covering the objectives during elective courses or post-graduate training. The objectives covered by respondents’ institutions included those pertaining to environmental health and sustainability of the workplace (e.g., general recycling procedures), research practice (e.g., sustainable laboratory practices), or the environmental impact of the healthcare system (e.g., waste production post patient care). Respondents stated that the objectives should be taught throughout medical education in an iterative format and noted that these objectives should be included in testing (e.g., standardized tests) to integrate and reinforce the importance of SHE education.

## Discussion

As human impact exerts pressure on the planet’s resources, the health of both ecosystems and humans are threatened. It is critical for learners to be conscious of, educated about, and responsive to this impact. Accordingly, we determined which SHE objectives should be taught when in the continuum of medical education. Overall our respondents indicated considerable agreement around which SHE objectives were important. Most objectives were considered important, with the objectives on the interaction between the environment and human health viewed as vital. Respondents noted that most, but not all, of the objectives should be covered primarily during the preclinical and clinical years of medical school. Based on the modified Delphi ratings, Table 3 displays the core SHE objectives and when each objective can be introduced during the continuum of medical education. This table serves as a guide for schools considering creating a SHE curriculum.

**Table 3.** Core set of objectives for teaching sustainable healthcare education (SHE) and timing in medical education during which to impart each objective as recommended by 52 experts during a modified Delphi procedure.

Domain	Doctor as scholar and scientist	Doctor as practitioner	Doctor as professional
Prior to medical school	–	Define environmental sustainability	–
Pre-clinical Years of medical school	- Outline the dependence of human health on global and local ecological systems, which supply essentials such as air, water, and a stable climate - Describe the mechanisms by which human health is affected by environmental change, for example through changes in disease vectors, exposure to extreme weather, migration, and reduced food security. - Describe features of a health-promoting local environment, in community and healthcare settings, to include access to green spaces, clean air and an active travel infrastructure - Discuss the contribution of human activity and population size to global environmental changes such as climate change, biodiversity loss and resource depletion - Explain the concept of environmental justice and the core principles for addressing it	- Identify potential synergies between policies and practices that promote environmental sustainability and those that promote health - Explain how trends in demographics, technology, climate, and resource availability may affect our ability to provide healthcare into the future	- Discuss ethical tensions between allocating resources to individual patients and protecting the environment upon which the health of the wider community depends - Explain how the health impacts of environmental change are distributed unequally within and between populations and the disparity between those most responsible and those most affected by change
Clinical years of medical school	- Discuss medical, ethical, legal, and economic factors in caring for patients with environmental disease	- Take a focused occupational and environmental history - Describe, with examples, the different types of environmental impact resulting from healthcare provision, and how these may be measured	- Evaluate their work environment for level of sustainability
Post-graduate years	–	- Identify ways to improve the environmental sustainability of health systems – in individual practice, in health service management, and in the design of care systems	–

Non-core objectives for inclusion:

Diagnose, treat, and/or prevent adverse health effects attributable to global climate change or environmental causes (e.g., illness from extreme weather conditions, disease vectors, inspired air pollutants - in particular ozone, particulate matter, and/or ingested pollutants from food or water). Explain bioaccumulation and biomagnification of pollutants Identify patients most vulnerable to climate change and advise them accordingly Discuss competing interests within healthcare (cost, infection control, safety) contributing to environmental inefficiency Recognize and articulate personal values concerning environmental sustainability, given the relationship between the environment and the health of current and future generations Demonstrate awareness of organizational sustainability policies and the legal frameworks for reducing carbon emissions

Most of the objectives on the survey were primarily knowledge-focused. Five of the objectives focused partially or completely on skills or attitudes related to SHE (i.e., take a focused occupational and environmental history, diagnose and prevent adverse health effects, identify patients most vulnerable to climate change, evaluate work for level of sustainability, recognize and articulate personal values). Of these five, consensus for inclusion was attained for two (i.e., take a focused occupational and environmental history and evaluate work for level of sustainability). The lower consensus for the skill/attitude objectives may have been because of the preponderance of knowledge objectives. However, SHE is in the early stages of discourse on development and inclusion. Hence it is likely that the prioritizing of knowledge-based objectives reflects the most immediate need to address the basics in lack of knowledge. The latter perspective is corroborated in recent work [32], in which Walpole and colleagues note that health professionals have basic SHE awareness but lack knowledge of its many aspects. Moreover, they also note that the attention given to SHE in medical education has been sparse.

We found that although SHE was covered at a few of the respondents’ institutions, the primary focus was on the sustainability-of-practice aspect of SHE (i.e., sustainability in the workplace, research and provision of healthcare) and was not always part of core education. These findings speak to the larger challenge of the crowded medical school curriculum faced by those of us considering the inclusion of SHE and those who seek to teach topics critical to evolving societal healthcare needs such as nutrition, violence prevention, and structural competency. Our study accounted for part of this challenge by prioritizing which objectives should be taught when. Recent discussion is beginning to address the next step in SHE curriculum development which points to a broad range of pedagogical approaches that may be used in SHE such as case-based, didactic, e-learning, and skills-based methods [32]. Ultimately, these suggestions do not provide a thorough solution to the problem of the overcrowded curriculum but serve to mitigate some of the overcrowding.

Future research should explore how institutions have chosen to implement objectives, instructional methods selected, and lessons learned. In addition, it will become essential to explore how institutions have secured support from the leadership or have leveraged existing structures to include SHE content in the curriculum.

Limitations of our study were that most of our respondents were from the United States. Our snowball sample was identified by less than half of the respondents in round 1, potentially skewing the perspectives offered. We limited our respondents to one option when rating the ‘timing in training’ question for each objective. We were seeking optimal time in training for each objective; however, allowing one option may have limited respondents from recommending all applicable time periods for each objective.

## Conclusion

Physicians, in their role as advocates, are accountable to society to improve the health of patients and communities [7]. This advocacy includes environmental accountability defined as the ‘obligation (of medical schools) within the social accountability framework to ensure their education, research, and service activities help to actively develop, promote, and protect environmentally sustainable solutions to address the health concerns of the community, region, and the nation that they have a mandate to serve’ [15]. A SHE curriculum places at the nexus of what physicians need to know the impact of the climate and environment on health as well as the impact of the healthcare system on the environment. Ultimately SHE education is vital as climate change and environmentally unsustainable practices pose perils to human health and existence. [1,33] Increased knowledge means environmentally sustainable practices are learned [12] and further environment-related deterioration of the health of society and planet, prevented.
